# Supplementary material for: Reconciling Mining with the Conservation of Cave Biodiversity: A Quantitative Baseline to Help Establish Conservation Priorities
Source: PLoS One. 2016 Dec 20;11(12):e0168348. doi: 10.1371/journal.pone.0168348 (PMC5173368; doi:10.1371/journal.pone.0168348)
Supplement: S1 Dataset — (ZIP) [file pone.0168348.s002.zip › Taxa/Serra Norte/SN_2013/N4WS/Lista N4WS-49.pdf]

CAVIDADE N4WS-0049

Classe	Ordem	Fam/Outros	Gên/Outros	Espécie	S-U	U-S	Zon.
Annelida	Oligochaeta			sp.		2	E
Arachnida	Acari	(Oribatida)		sp.2		X	E/P
Arachnida	Acari	Rhagidiidae		sp.1	X		
Arachnida	Araneae	Araneidae		jovem	X		E
Arachnida	Araneae	Ctenidae		jovem	1		E
Arachnida	Araneae	Mysmenidae		jovem		X	E
Arachnida	Araneae	Ochyroceratidae	*Speocera*	sp.1	X		E
Arachnida	Araneae	Pholcidae	*Mesabolivar*	*eberhard*		X	E
Arachnida	Araneae	Scytodidae	*Scytodes*	*globula*	9	1	E
Arachnida	Araneae	Theridiosomatidae	*Plato*	sp.1	X		E
Arachnida	Araneae	Thomisidae		jovem	1		E
Arachnida	Opiliones	Sclerosomatidae	*Prionostema*	sp.1	X	X	E
Arachnida	Pseudoscorpiones	Chernetidae	*Spelaeochernes*	sp.1	X	X	E/P
Arachnida	Pseudoscorpiones	Chthoniidae	*Pseudochthonius*	sp.1	X		E
Diplopoda	Polydesmida	Chelodesmidae		sp.1		1	E
Entognatha	Collembola	Paronellidae		sp.1	X		E
Entognatha	Collembola	Paronellidae		sp.6		X	E
Entognatha	Collembola	Sminthuroidea		sp.1	X		E
Insecta	Blattodea			jovem	2		E
Insecta	Coleoptera	Nitidulidae		sp.1	X		E
Insecta	Coleoptera	Scarabaeidae		jovem	X		E
Insecta	Diptera	Cecidomyiidae	Cecidomyiinae	sp.	X		E
Insecta	Diptera	Ceratopogonidae		jovem	X		E
Insecta	Diptera	Phoridae	Metopininae	sp.	X		E
Insecta	Diptera	Psychodidae	*Psychodopygus*	Série *chagasi*		X	E
Insecta	Diptera	Psychodidae	*Sciopemyia*	*sordellii*	X	X	E/P
Insecta	Diptera	Sciaridae	*Pseudosciara*	sp.	X		E
Insecta	Diptera	Tipulidae	Tipulinae	sp.	X		E
Insecta	Heteroptera	Cydnidae	*Pangaeus*	sp.1		X	P
Insecta	Heteroptera	Hebridae	Hebrinae	sp.	X		E
Insecta	Heteroptera	Ochteridae		sp.	X		E
Insecta	Heteroptera	Reduviidae	Emesinae	jovem		1	P
Insecta	Hymenoptera	Formicidae	*Acropyga*	sp.1		X	P
Insecta	Hymenoptera	Formicidae	*Apterostigma*	sp.1	X		E
Insecta	Hymenoptera	Formicidae	*Gnamptogenys*	*striatula*		X	E
Insecta	Hymenoptera	Formicidae	*Nylanderia*	sp.1	X		E
Insecta	Hymenoptera	Formicidae	*Pheidole*	sp.2	X		E
Insecta	Orthoptera	Phalangopsidae	*Paraclodes*	sp.	1		E
Insecta	Orthoptera	Phalangopsidae	*Phalangopsis*	sp.	30		E
Insecta	Psocoptera	Psocomorpha		jovem		X	E
Symphyla		Scutigerellidae	*Hanseniella*	sp.1	X	X	E/P
Amphibia	Anura	Strabomantidae	*Pristimantis*	cf. *fenestratus*	2	1	E/P
Mammalia	Chiroptera	Emballonuridae	*Peropteryx*	sp.	5	2	E
Mammalia	Chiroptera	Phyllostomidae	*Carollia*	*perspicillata*		2	
Reptilia	Squamata	Boidae	*Epicrates*	sp.	1		E
